# Murine model for chronic rhinosinusitis: an interventional study

**DOI:** 10.1186/s40463-023-00637-6

**Published:** 2023-04-25

**Authors:** Amr F. Hamour, John JW. Lee, Ewa Wasilewski, Eric Monteiro, John M. Lee, Allan Vescan, Lakshmi P. Kotra

**Affiliations:** 1 Department of Otolaryngology – Head and Neck Surgery, Temerty Faculty of Medicine, University of Toronto, Toronto, ON Canada; 2 Centre for Cannabinoid Therapeutics and Centre for Molecular Design and Preformulations, Krembil Research Institute, University Health Network, Toronto, ON Canada; 3 Department of Otolaryngology – Head and Neck Surgery, Sinai Health System, Toronto, ON Canada; 4 Department of Otolaryngology – Head and Neck Surgery, St. Michael’s Hospital, Toronto, ON Canada; 5 Department of Pharmaceutical Sciences, Leslie Dan Faculty of Pharmacy, University of Toronto, Toronto, ON Canada

**Keywords:** Animal model, Chronic rhinosinusitis, Eosinophilic chronic rhinosinusitis, Histological markers, Inflammation

## Abstract

**Background:**

Chronic rhinosinusitis (CRS) is a complex inflammatory disease of the sinonasal tract. To understand this disease entity and develop targeted treatments, a reproducible animal model is paramount.

**Aims/objectives:**

To optimize a murine model of eosinophilic CRS by establishing benchmark histological markers and validate its fidelity in evaluating intranasal treatments.

**Material and methods:**

Forty-five Balb/c mice were included in the 7-week protocol. Experimental animals (n = 20) were induced a CRS disease state upon receiving intraperitoneal sensitization with ovalbumin (OVA), followed by intranasal OVA with *Aspergillus oryzae* protease. Analysis of complete blood count with differential, peripheral blood smear, and histological markers from the nasal cavity mucosa were performed. CRS mice were additionally treated with intranasal saline (n = 5) or mometasone (n = 10) and compared with control groups of untreated CRS (n = 5) and healthy (n = 5) mice after week 7.

**Results:**

Histological analysis of experimental animal nasal mucosa revealed significantly higher levels of eosinophilic tissue infiltration/degranulation, hyaline droplets, Charcot–Leyden crystals, and respiratory epithelial thickness compared to healthy controls. Treatment with mometasone significantly reversed the histopathological changes observed in CRS mice.

**Conclusion and significance:**

This murine model induced substantial local eosinophilic inflammation within sinonasal mucosa, that was reversible with mometasone. This model may be used to evaluate the efficacy of therapeutics designed to target CRS.

## Introduction

Chronic rhinosinusitis (CRS) is a complex inflammatory disease of the sinonasal cavity that has a poorly understood etiology. The negative impact of CRS on patients’ overall quality of life and work productivity is well documented. In the United States, current estimates of prevalence of CRS vary between 2.3 and 12.1%. The overall direct cost related to CRS is estimated to range between $10 and $13 billion per year [1]. Given such a significant impact on individual patients and society at large, understanding the cellular basis of this disease entity and developing reliable treatment options is paramount.

Conventional management for CRS involves a combination of topical and systemic medical therapy, with or without the need for surgery. The initiation of a treatment plan often hinges on the phenotype of CRS the patient presents with, traditionally categorized as CRS with polyposis (CRSwNP) or CRS without polyposis (CRSsNP) [2]. CRS is further subdivided into type 2 and non-type 2 dominant disease [3]. Type 2 endotype disease encompasses CRSwNP and eosinophilic CRS. Currently, multiple therapies are used in the treatment of CRS including saline rinses and sprays, intranasal and systemic glucocorticoids, antibiotics, and anti-leukotriene agents [3]. Despite the evolving treatment options available, many patients continue to fail conventional therapy. To better understand the pathophysiology of CRS and subsequently develop novel immunomodulatory treatment options, we must first have a physiological understanding of his complex disease.

In vivo models serve as useful tools for understanding the pathogenesis and pathophysiology of disease. Several studies have evaluated animal models for CRS, including mouse, rabbit, and sheep models [4, 5]. Given the heterogeneity in the suspected pathophysiology of CRS phenotypes, developing an all-encompassing animal model can be very challenging. Type 2 endotype dominant disease, and more specifically eosinophilic CRS, is distinguished by nasal mucosal accumulation of eosinophils and a predominantly Th2 cytokine response [6, 7]. Clinically, these patients can be challenging to manage as their disease can be recalcitrant to traditional therapy [2, 3]. Therefore, having a reliable animal model that represents the type 2 disease process and enables testing of therapeutics is imperative. A murine model of allergic CRS representing eosinophilic sinonasal inflammation has been previously described [7]. Advantages of a murine model include cost-effectiveness, ease of animal handling, and the ability to scale experiments to large cohorts. The aim of this study was to establish benchmark histological markers of sinonasal inflammation for a murine model of eosinophillic CRS. Secondly, we aimed to establish its fidelity in evaluating therapeutics used in the management of CRS, such as nasal saline and intranasal corticosteroids.

## Methods

### Experimental animals

The study protocol was approved by the Animal Care Committee at the University Health Network (Toronto, Ontario, Canada). Forty-five six-week-old Balb/c (Jackson Laboratory, Bar Harbour ME) were utilized in the protocol as described below. Body weight and general health were monitored (5 days/week) with the following observations monitored: respiration rate, allergic reactions (visual inspection of the nose, eyes, mouth, fur), grooming behaviour, generalized weakness, hydration level, abnormal behaviour, and posture.

### Murine model protocol and animal handling

**Fig. 1:**
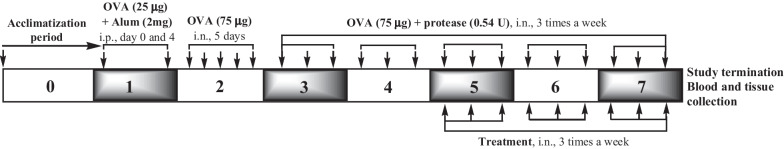
Protocol for CRS murine model induction and intranasal treatments. *i.p.* intraperitoneal, *i.n.* intranasal

A previously established allergic rhinosinusitis murine model was adapted with modifications for the experimental protocol [7]. Induction of CRS in 20 Balb/c mice (10 females and 10 males) was performed using ovalbumin (OVA) and *A. oryzae* protease (Fig. 1). The CRS model induction started with intraperitoneal injection of 25 µg of OVA and 2 mg of Alum (Aluminum hydroxide adjuvant) on day 0 and 4 (Week 1). In the second week, mice received intranasal rinse with 75 µg of OVA in 30 µL of PBS for 5 consecutive days (Mon–Fri). In weeks 3–7, mice received intranasal rinse contained 75 µg of OVA and 0.54U of *A. oryzae* protease. The dosing was done three times a week. Control mice (n = 10) were administered PBS. At the end of week 3 to 7, six mice (3 males and 3 females) were euthanized, and blood and tissue were collected for analysis. An additional fifteen CRS mice (n = 15) were treated with intranasal saline (n = 5) or mometasone (n = 10). The treatments were performed three times a week for three weeks (week 5–7), as determined by resource allotment and availability of study personnel. The concentration of mometasone was 0.5 mg/ml with 0.02 mg (1 mg/kg) delivered each dose, in line with intranasal doses reported in other mouse models in the literature (0.01–3 mg/kg) [8, 9]. Mice were euthanized for sample collection and analysis at week 7. Control groups of untreated CRS mice (n = 5) and healthy mice (n = 5) were compared after week 7.

**Fig. 2:**
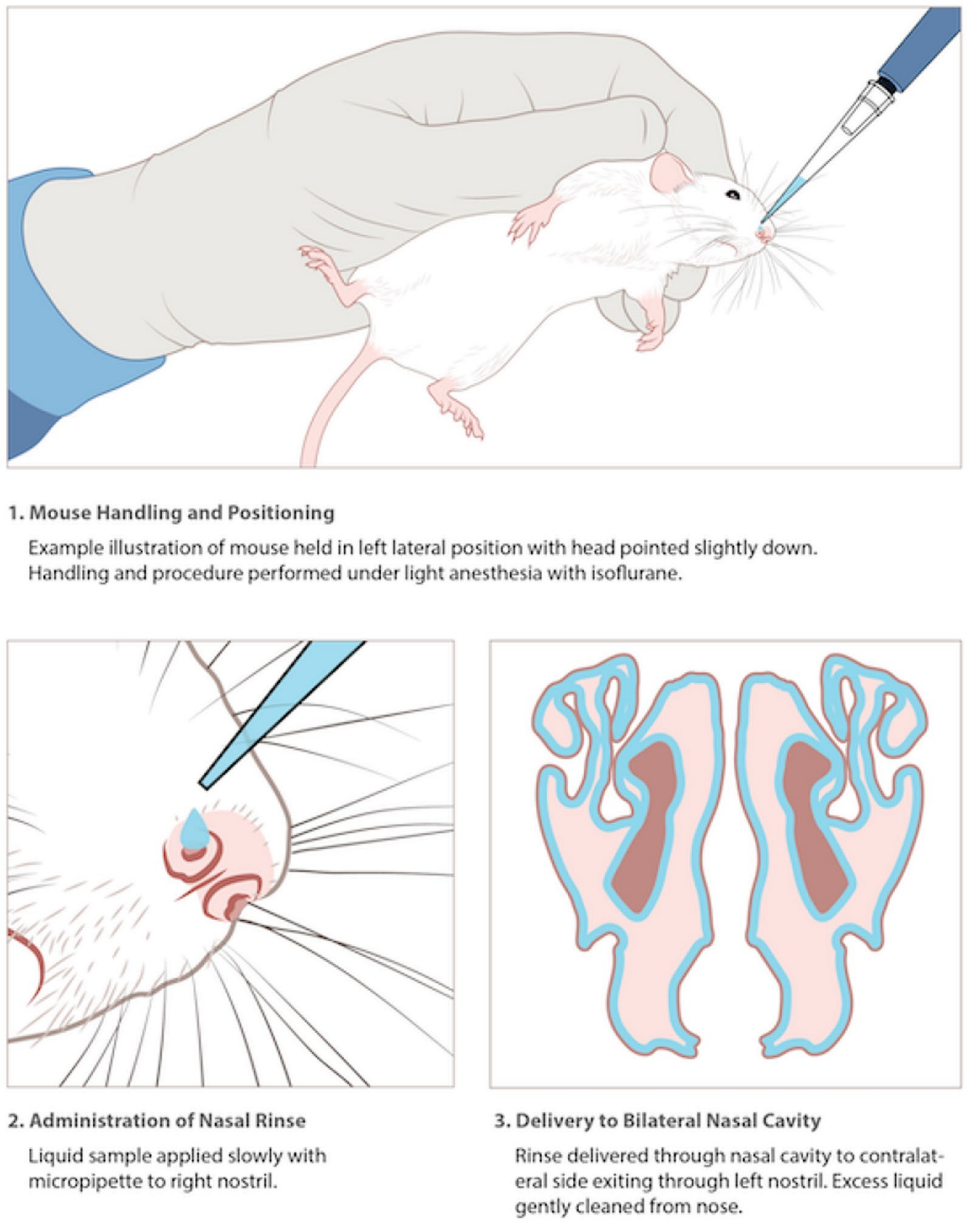
Handling of mice and method of intranasal administration

Administration of intranasal induction and treatment was done via nasal rinse done under light anesthesia (isoflurane, 5% induction, 2% maintenance) (Fig. 2). Mice were held in a left lateral position with the head pointed very slightly down and the liquid sample (30 µL) was applied slowly to the right nostril. The sample traveled through the nasal cavity and exited the nose through the left nostril. Usually, once a drop exited the left nostril it was gently wiped away to draw the excess of liquid from the nose. The procedure worked as a rinse and was done in a manner to avoid any liquid being drawn into the lungs.

### Histological analysis

Mice were euthanized, with head specimens initially fixed in 10% formalin. Tissue samples were further processed, sectioned and stained at the Toronto Centre for Phenogenomics (Toronto, Canada). Nasal cavity sections with a thickness of 4 μm were stained with Hematoxylin and Eosin (H&E) to study the tissue morphology and to evaluate the symptoms of inflammation. Periodic acid-Schiff stain was used to view goblet cells. Stained sections were scanned at 20× magnification.

Respiratory epithelium was evaluated in various areas around the nasal septum, vomeronasal organ (VNO), and the turbinates (maxilloturbinates). The infiltration of eosinophils was evaluated by counting the eosinophils in H&E stained sections and recorded as cells per mm [2]. Respiratory epithelial thickness was measured as the distance between the apex of the epithelial cells and the upper border of the subepithelial glands zone. Fifteen measurements were taken for each specimen (three cross sections, 5 measurements each). The following inflammation markers were evaluated as previously described with scoring performed blinded to study groups: degranulation of eosinophils, the presence and amount of hyaline droplet material, and the occurrence of eosinophilic crystals (Charcot–Leyden crystals) [8]. Scores from 0 to 4 were used to describe the severity of the markers. (0) none, (1) minimal, (2) mild, (3) moderate, and (4) severe. Minimal was defined as barely detectable, mild as slightly detectable, moderate as easily detectable, and severe as very evident. Whole blood was also collected vial cardiac puncture and used for CBC analysis (lymphocytes, monocytes, neutrophils) and to prepare blood smear samples (eosinophils).

### Statistical analysis

Data values were expressed in mean and standard deviation. Independent *t*-test was used to compare groups. P-value threshold of < 0.05 was used to indicate statistical significance. Statistical analyses were performed using SPSS (Version 23, IBM Corp., Armonk, NK.). Data plotting was performed using Microsoft Excel (Version 16.52, Redmon WA).

## Results

### Inflammatory markers of chronic rhinosinusitis

**Fig. 3:**
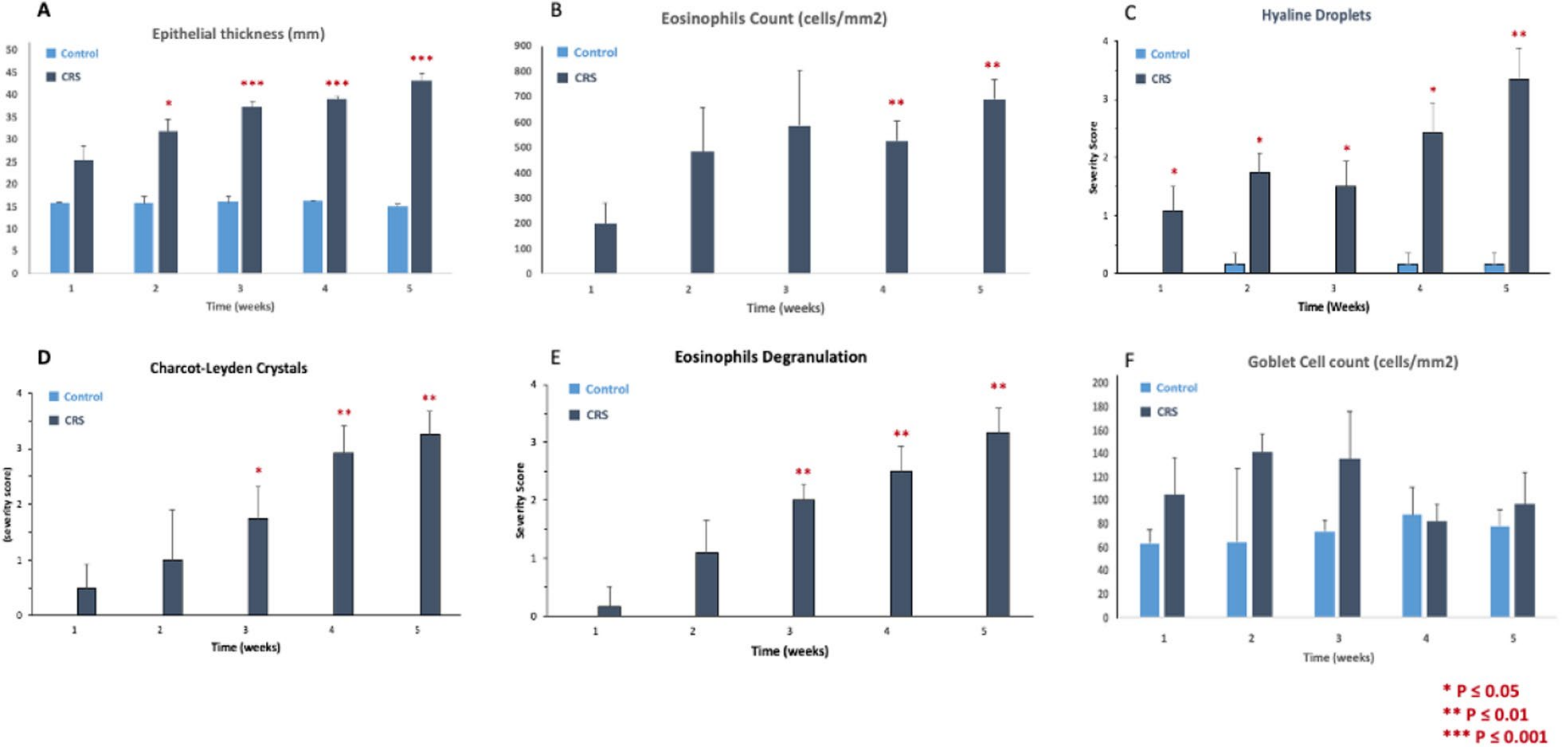
Histopathological markers of chronic sinonasal inflammation in CRS and healthy mice. Comparison of histopathological characteristics representing chronic sinonasal inflammation (epithelial thickness, eosinophils count, hyaline droplets, Charcot–Leyden crystals, eosinophil degranulation and goblet cell count) between experimental and control mice. Analysis was performed at the end of week 3 to 7. P-value thresholds are indicated by *P < 0.05, **P < 0.01, and ***P < 0.001

**Fig. 4:**
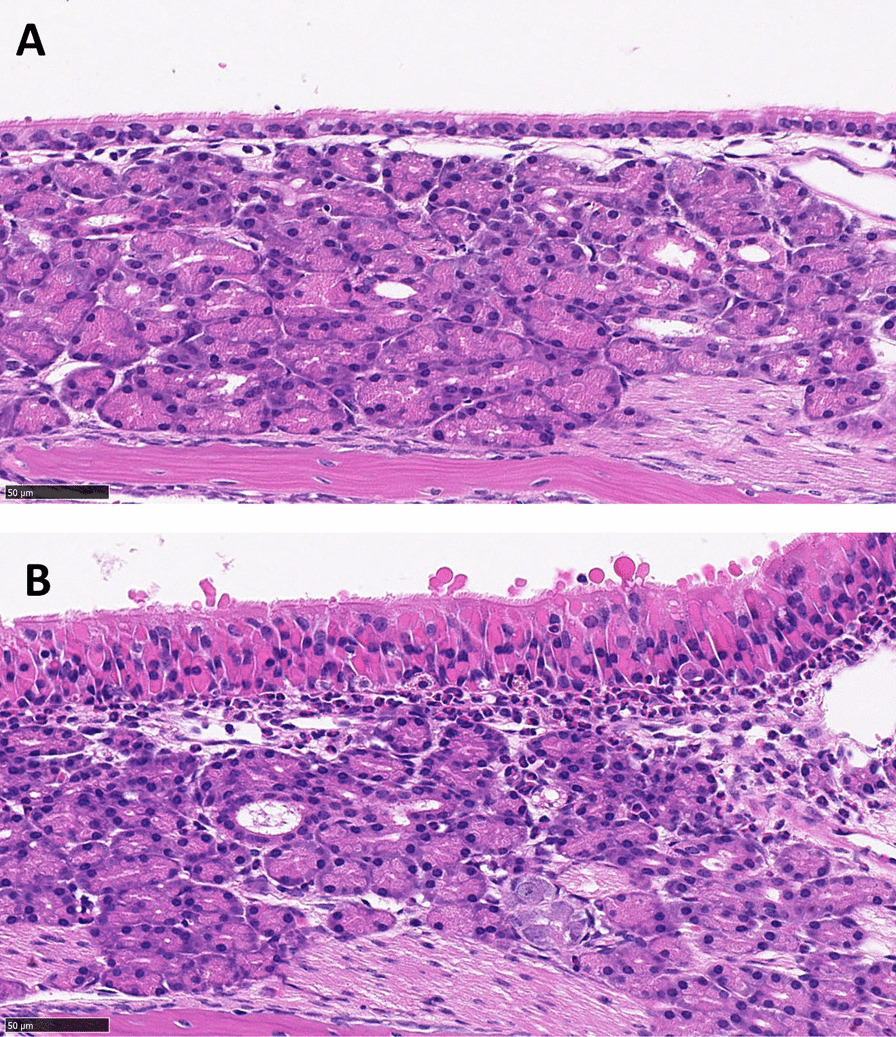
Respiratory nasal mucosa of CRS and healthy control mice. Hematoxylin and eosin stain at ×40 magnification of the nasal respiratory mucosa is presented in **A** Healthy Control and **B** CRS mice after ovalbumin/protease induction. In the CRS mice, the epithelium is hyperplastic with increased thickness of the lamina propria observed. Increased eosinophils and degranulation are also seen in the subepithelial layer in CRS mice compared to healthy controls

Histopathological markers of sinonasal inflammation representative of CRS are shown quantitatively over the study period in Fig. 3. Presence of eosinophils, Charcot–Leyden crystals, hyaline droplets and eosinophilic degranulation was observed in only CRS induced mice treated with OVA/protease compared to control mice (Fig. 3A–E; p < 0.05 for all). Epithelial thickness was significantly greater in the CRS group versus controls (p < 0.001). No significant difference was observed in goblet cell counts between the two groups. Histological evaluation of respiratory nasal mucosa showed hyperplastic epithelium with increased thickness of the lamina propria as well as increased eosinophils and degranulation in the subepithelial layer in OVA/protease induced CRS mice compared to healthy controls (Fig. 4).

### Intranasal treatment with saline and mometasone in CRS mice

**Fig. 5:**
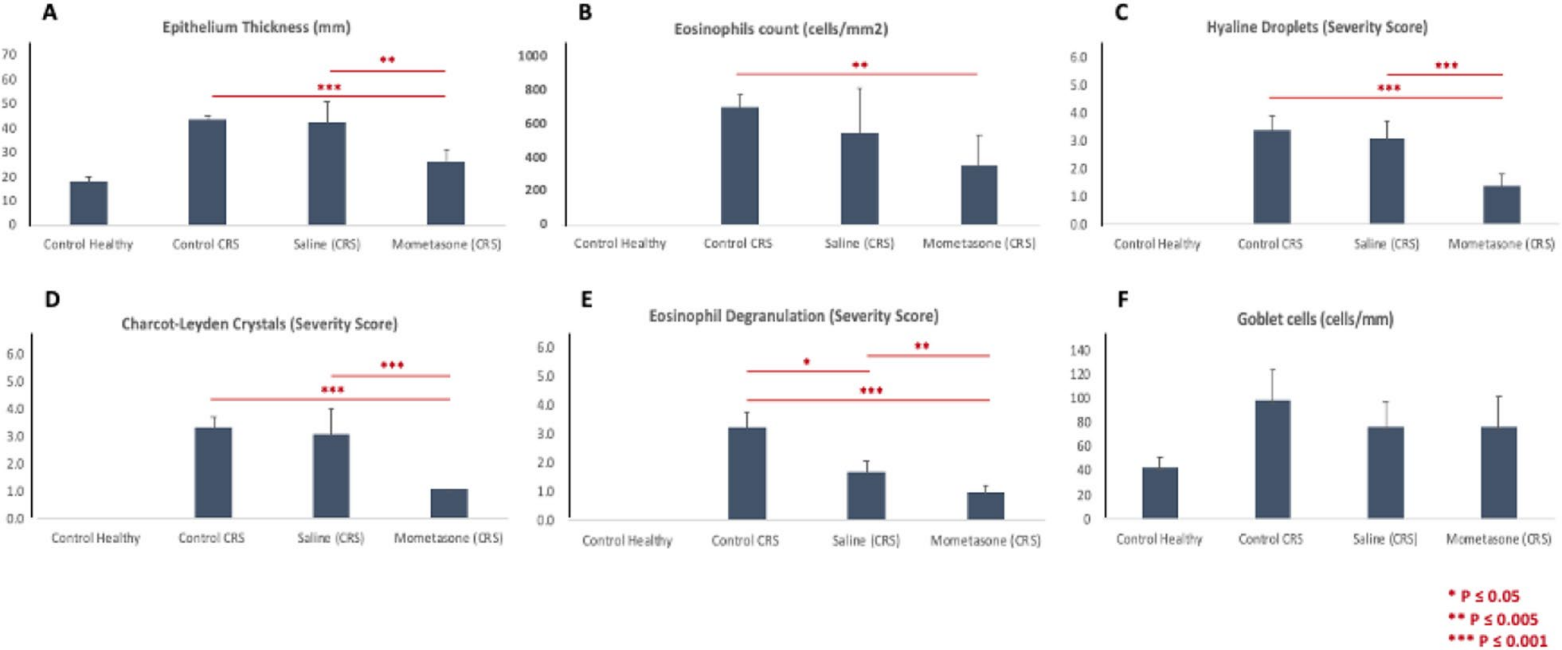
Comparison of inflammatory markers in CRS mice treated with intranasal saline and mometasone. Comparison of histopathological characteristics representing chronic sinonasal inflammation (epithelial thickness, eosinophils count, hyaline droplets, Charcot–Leyden crystals, eosinophil degranulation and goblet cell count) between healthy controls, control CRS (untreated), Saline treated CRS, and mometasone treated CRS mice Analysis was performed at the end of week 7. P-value thresholds are indicated by *P < 0.05, **P < 0.01, and ***P < 0.001

**Fig. 6:**
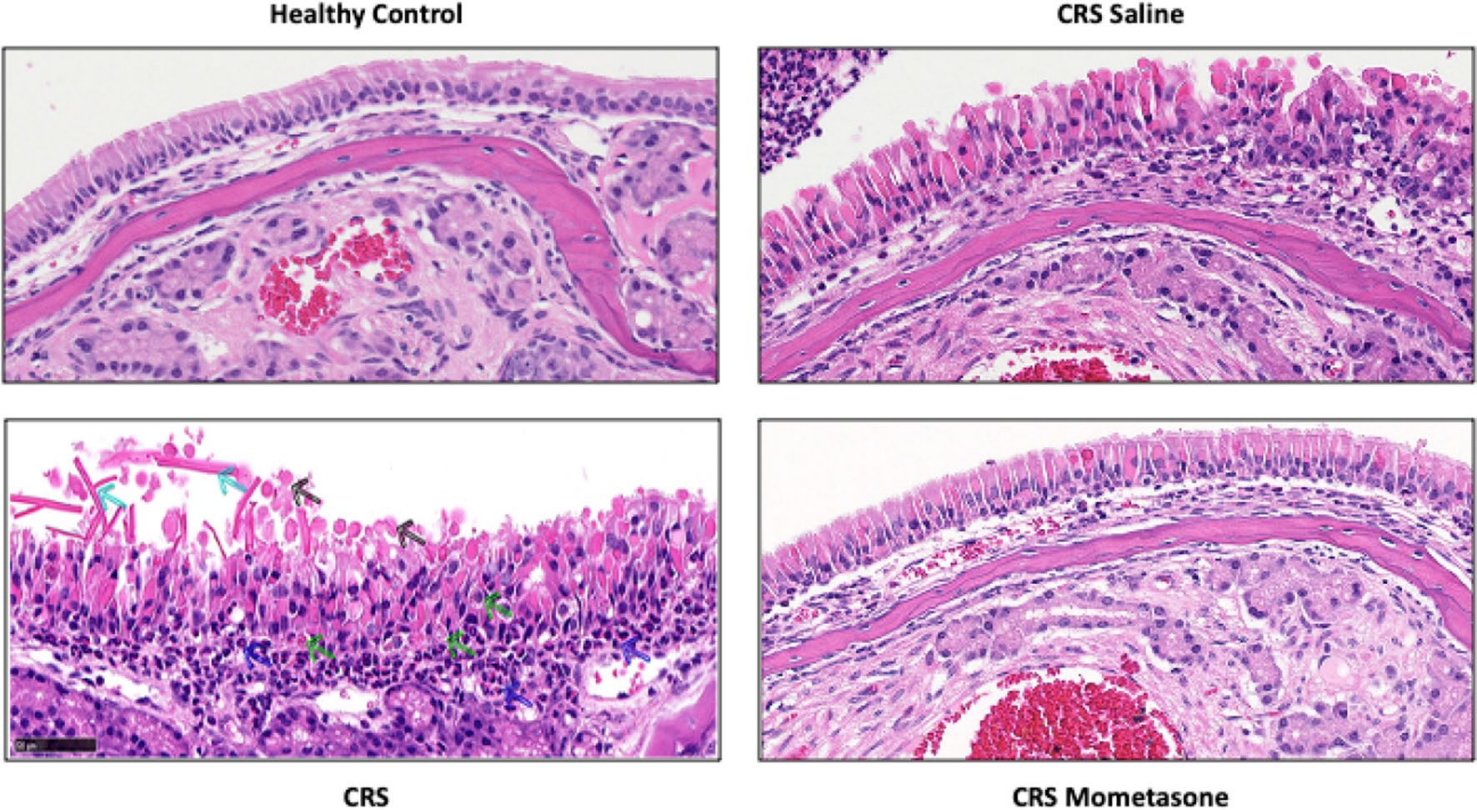
Respiratory epithelium of healthy mice, CRS mice, saline and mometasone treated CRS mice. Hematoxylin and eosin stain at ×40 magnification of the nasal respiratory mucosa is presented in healthy control, CRS untreated, CRS saline treated and CRS mometasone treated mice. Blue arrows indicate eosinophils, (green) degranulating eosinophils, (black) Hyaline material discharged from the epithelium layer, (cyan) Charcot–Leyden crystals

Inflammatory markers of CRS were compared between healthy, untreated CRS and CRS mice treated with intranasal saline or mometasone (3 times a week for 3 weeks) (Fig. 5). Epithelial thickness, eosinophilic count and degranulation, hyaline droplets and Charcot–Leyden crystals were significantly reduced in CRS mice treated with mometasone compared to untreated CRS mice (Fig. 5A–E; p < 0.005 for all). Treatment with intranasal saline demonstrated an improvement in eosinophilic degranulation alone (Fig. 5E). Compared to intranasal saline, mometasone treated CRS mice had significantly reduced epithelial thickness, hyaline droplets, Charcot–Leyden crystals and eosinophilic degranulation. No differences in goblet cell count were observed between groups (Fig. 5F). Overall, respiratory epithelium of CRS mice treated with intranasal mometasone demonstrated a general reversal of inflammatory markers compared to untreated and saline treated CRS mice (Fig. 6).

### Safety

No study animals died during the induction and treatment periods of the study protocol. OVA/protease treated CRS mice did not demonstrate any signs of abnormal behaviour, allergic reaction, atypical respiration, or generalized malaise throughout the study. Average body weight measured throughout the study period was similar between the two groups. No significant differences were observed in the weekly CBC differential and blood smear analysis between healthy control and CRS mice.

## Discussion

In this study, we successfully establish the fidelity of a murine model of CRS to reliably evaluate intranasal therapeutic interventions. Specifically, multiple inflammatory markers characteristic of chronic sinonasal inflammation were validated as sensitive quantitative outcomes to represent CRS. More importantly, intranasal treatment with mometasone reversed mucosal histological changes associated with eosinophilic CRS. No morbidity or mortality were observed in the study, reinforcing the safety of the model.

In vivo animal models of sinusitis have been previously described in mice, rabbits, and sheep [4]. Though the sinus anatomy of rabbits shares the most similarities with humans, a murine model offers multiple advantages including greater capability for histopathological and immunochemical analyses and significantly lower costs allowing for scaling of experiments [5]. Several topical treatments have been evaluated using animal models, including colloid silver, tofacinib, and tobramycin [10–12]. Previous studies of murine models have evaluated histological features such as eosinophil levels, subepithelial collagen deposition, mast cell and goblet cell count to characterize chronic sinusitis [7, 13, 14]. Our study validates several additional markers of allergic sinonasal inflammation to serve as benchmark measurements of CRS. Quantification of eosinophilic degranulation more accurately represents its activation and infiltration, while Charcot–Leyden crystals are considered key markers of eosinophilic inflammation that have been shown to be predictive of recurrent CRS disease in humans [15, 16]. Furthermore, thickening of the epithelial and subepithelial layers of the nasal respiratory mucosa is demonstrated in the mucosal remodelling of CRS [17].

In our study, treatment with intranasal mometasone demonstrated reversal of several histopathological findings of eosinophilic inflammation. This panel of inflammatory markers both reflects the general anti-inflammatory properties of corticosteroids, but more importantly, showcases that the disease state induced in our model is reversible. Intranasal corticosteroids have been the mainstay for treatment in CRS for decades [3]. Their primary mechanism of action is via the cytoplasmic glucocorticoid receptor (GR) [18]. Modifications to gene transcription via trans-activation or trans-repression result in a wide-ranging downstream anti-inflammatory cascade [18]. The histological results of mometasone-treated mice in our study illustrate the cellular outcomes of this cascade.

The utility of a reproducible and reliable animal model for disease cannot be overstated. Given the nature of CRS being a mucosal disease, treatment has largely resolved around topical administration of anti-inflammatory medication. In vitro studies of cultured nasal tissue have served as a useful tool in testing such medications [19, 20], however, this in vivo animal model enables us to evaluate how topical medications in the sinonasal cavity may impact the overall health of the animal. This is valuable for future applications as it can allow for the testing of novel anti-inflammatory or immunomodulatory topical agents prior to proposed administration in humans. Given the ability to readily evaluate markers for inflammation, the direct impact of such interventions can be assessed to shed light on pathophysiological implications of potential therapeutics.

This study has some notable limitations. We did not perform immunochemical analyses to evaluate inflammatory cytokines and antigens as demonstrated in previous studies [7, 14, 21]. Given the local nature of CRS, identifying potential inflammatory markers within sinonasal fluid may be instructive in grading the efficacy of potential therapeutics. With the establishment of a base model, developing and implementing assays for such analyses will be a beneficial addition to this protocol in the future. Secondly, our study may not translate to other CRS phenotypes such as CRSwNP. Although this study protocol induces eosinophilic CRS, it does not necessarily induce polyp formation. Murine models representing sinonasal inflammation with nasal polyposis have been previously reported in literature using *staphylococcus aureus* enterotoxin B [22]. Recent studies investigating intranasal cyclosporine and tofacitinib in a nasal polyposis model utilized intranasal triamcinolone as a treatment control, however the reversal of polyp formation was shown to be inconsistent between studies [11, 23]. Lastly, although there is histological concordance between experimental animal sinonasal epithelium in this study and eosinophilic CRS in human sinonasal epithelium, this does not reflect symptomatology. CRS is a disease characterized by quality-of-life impairment, which may not align with clinical histological, or pathological findings. As such, an animal model is limited in that it does not provide a direct corollary to both the disease process in humans and the symptomatology associated with it.

## Conclusion

In this study, a murine model of eosinophilic CRS, utilizing OVA with *Aspergillus oryzae* protease, induced substantial local eosinophilic inflammation within sinonasal mucosa. Moreover, treatment with intranasal mometasone led to partial reversal of local inflammation. In the future, this model may be used to reliably evaluate the efficacy of therapeutics designed to target CRS.

## Data Availability

The datasets used and/or analysed during the current study are available from the corresponding author on reasonable request.
